# Parental Risk Factors and Child Birth Data in a Matched Year and Sex Group Cleft Population: A Case-Control Study

**DOI:** 10.3390/ijerph18094615

**Published:** 2021-04-27

**Authors:** Inês Francisco, Francisco Caramelo, Maria Helena Fernandes, Francisco Vale

**Affiliations:** 1 Institute of Orthodontics, Faculty of Medicine, University of Coimbra, 3000-075 Coimbra, Portugal; 2 Institute of Clinical and Biomedical Research of Coimbra (iCBR), Faculty of Medicine, University of Coimbra, 3000-075 Coimbra, Portugal; fcaramelo@fmed.uc.pt; 3 Faculty of Dental Medicine, University of Porto, 4200-393 Porto, Portugal; mhfernandes@fmd.up.pt; 4 LAQV/REQUIMTE, University of Porto, 4160-007 Porto, Portugal

**Keywords:** cleft palate, cleft lip, environmental risk factors, parental age, body mass index, child birth data

## Abstract

(1) Background: The etiology of orofacial cleft (OC) is not completely known but several genetic and environmental risk factors have been identified. Moreover, a knowledge gap still persists regarding neonatal characteristics. This study evaluated the effect of parental age and mothers’ body mass index on the risk of having an OC child, in a matched year and sex group (cleft/healthy control). Additionally, birth data were analyzed between groups. (2) Methods: 266 individuals born between 1995 to 2015 were evaluated: 133 OC individuals (85 males/48 females) and 133 control (85 males/48 females). A logistic model was used for the independent variables. ANOVA or Kruskal-Wallis tests were used for comparison between the OC phenotypes. (3) Results: Regarding statistically significant parental related factors, the probability of having a cleft child decreases for each maternal year increase (odds ratio = 0.903) and increases for each body mass index unit (kg/m^2^) increase (odds ratio = 1.14). On the child data birth, for each mass unit (kg) increase, the probability of having a cleft child decrease (odds ratio = 0.435). (4) Conclusions: In this study, only maternal body mass index and maternal age found statistical differences in the risk of having a cleft child. In the children’s initial data, the cleft group found a higher risk of having a lower birth weight but no relation was found regarding length and head circumference.

## 1. Introduction

Orofacial clefts (OC) are one of the most common craniofacial malformations, with an international prevalence in newborns of 14 per 10,000 live births worldwide [1,2]. The prevalence of OC has been increasing over the years, perhaps as a result of improved surgical techniques, neonatal care resulting in reduced postnatal morbidity and mortality, more accurate documentation, and environmental factors such as smoking consumption [2,3,4]. According to the European Network for Epidemiological Surveillance of Congenital Anomalies (EUROCAT) report, the prevalence in 26 European countries was 14.5 per 10,000 births between 2011 and 2018 [5].

The etiology of OC is not completely known but several genetic and environmental risk factors have been identified. Previous studies have clarified the importance of epidemiologic knowledge, especially in (1) assessing the burden of OC in order to plan public health resources; (2) knowing the causes of OC, which stimulates research on primary prevention and treatments to improve the quality of care [6]. The genetic component plays an important role in the formation of isolated cleft. Some of the best-supported genes and genetic loci in the literature are IRF6, ch8q24, VAX1, and PAX7 [7,8]. Concerning environmental risk factors, previous researches, which have shown consistency, have proposed smoking consumption during pregnancy and gestational diabetes as maternal risk factors [4,9,10]. Other associations have been reported with less consistency, namely alcohol consumption during pregnancy, intrapartium interval, maternal and paternal age, and nutritional deficiencies (folic acid, vitamin A and B12, and zinc) [11,12,13,14,15,16,17,18]. Furthermore, both extremes of maternal body mass index (BMI) have been associated with birth defects, but with controversial results in the literature [10,19,20,21]. Recently, Kutbi et al. showed in a larger population-based study that obesity was associated with an increased risk of OC but no conclusion was drawn about maternal underweight [21].

Even though there are several studies about OC etiological factors in the literature, there is a gap regarding neonatal characteristics. Several studies showed that OC children are lighter and smaller than healthy controls but with inconsistent results [22,23]. Despite the growing literature about cleft etiology, the main limitation of these studies is the recruitment process, since there is often heterogeneity in relation to the sociodemographic factors and sex and age of patients evaluated. This approach is expected to produce more bias since it is already known that some of these factors are determinants of laterality and side of the cleft, such as sex [24]. Furthermore, the studies published on cleft birth data are focused on weight, leaving aside some anthropometric measurements (e.g., length and head circumference at birth) that are used in the evaluation of intrauterine growth deviations, overall health of the child, and early and long-term growth [22,25,26].

This study aims to evaluate the effect of possible parental related influencing factors on the development of cleft lip and/or palate as well as the differences in birth data. Therefore, this study used child recruitment with standardized methods in order to obtain a homogeneous sample regarding age, sex, and sociodemographic factors. Moreover, length and head circumference at birth will be measured in addition to birth weight. The purpose of this study was to test the hypothesis that the risk of having a cleft child increased with parental age or maternal BMI, and also to compare the differences in birth data (birth weight, birth length, and head circumference at birth) between cleft children and healthy controls (non-cleft group).

## 2. Materials and Methods

### 2.1. Study Design

This retrospective case-control study was approved by the Ethics Committee of the Faculty of Medicine of University of Coimbra (Reference: CE-072/2020). The study was handled in accordance with the Declaration of Helsinki.

### 2.2. Data Collection Procedure

The study group included parents from children (born between 1995–2015), with non-syndromic OC, being rehabilitated at the Institute of Orthodontics, Faculty of Medicine, University of Coimbra (FMUC), regardless of sex or place of birth. The control group included individuals without clinical alterations or craniofacial defects. To minimize bias, controls were matched according to year of birth and sex. Both groups had similar socio-economic backgrounds.

Data were collected through two sources: (1) medically recorded data—parents were requested to send the pre-natal care logbook and all medical record during the pregnancy; (2) maternal self-reported information.

A structured questionnaire was also distributed to the parents of both groups after the clinical visit, avoiding any harm to the patients. The questionnaires were deployed always by the same examiner (I.F.) after being properly trained for the activity. Before fulfilling the questionnaire, parents were asked to read the enclosed list as a memory aid with the most common maternal diseases during pregnancy and pregnancy supplements. From both groups, the parents who did not consent to participate in the study or who did not answer the questionnaire sufficiently, parents with consanguineous marriages, and children with syndromic OC were excluded.

The questionnaire included retrospective parent and patient information. Variables were divided in three groups:

- Sociodemographic—age of parents and child; child’s sex.
- Obstetrical data—alcohol intake, smoking, drugs, and pregnancy supplements used, diseases diagnosed before and during pregnancy and exposure to carbon monoxide, recorded by gestational month; early pregnancy body mass index.
- Child birth data—presence and phenotype of cleft, birth weight, birth length, and head circumference at birth.

**Figure 1:**
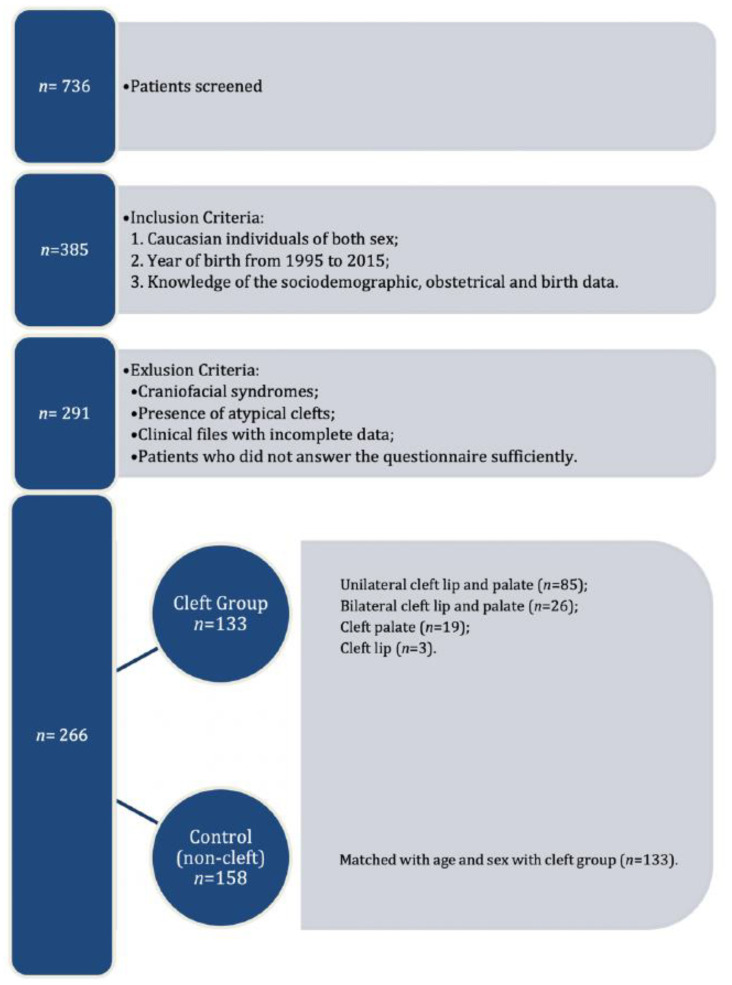
Flow chart of the inclusion and exclusion criteria.

After matching the control group to the OC group, the final sample consisted of 266 individuals (Figure 1). All participants gave their written informed consent for secondary use of their records.

### 2.3. Statistical Analysis

All analyses were performed using the Statistical Package for the Social Sciences, version 26.0 for Windows (SPSS Inc., Chicago, IL, USA). The significance level chosen was 0.05.

Descriptive statistics for the quantitative variables were obtained using mean, standard deviation values, and 75th and 25th percentiles. Categorical variables were expressed as absolute and relative frequency.

The comparison between the groups was performed using Fisher’s exact test for gender (nominal variable), and for the quantitative variables, the Mann-Whitney test was used when a violation of the normality assumption was verified through the Shapiro-Wilk test.

To assess the risk factors, a logistic model was adjusted using as independent variables parental age, maternal BMI, and birth data (weight, length, and head circumference at birth). The logistic model was evaluated using the Naguelkerke’s R2 and by measuring the area under the ROC curve. The null hypothesis of the statistical analysis (the logistic regression) is that no risk factors exist.

Comparison between the different phenotypes of cleft was performed using the ANOVA test or the Kruskal-Wallis test when the normality assumption was not observed. Tukey’s post-hoc tests were performed, after verifying the significant ANOVA test.

## 3. Results

In total, 266 eligible cases were identified in the FMUC between 1995 and 2015, among them 133 were orofacial cleft and 133 were control group.

Table 1 presents descriptive statistics concerning sex distribution, parental age, maternal BMI, and birth data of the subjects studied. The distribution of age and sex is homogenous (*p* = 1.00) between groups (Control vs. Orofacial Cleft).

Three independent variables (maternal age, maternal BMI, and child birth weight) found statistical significance using the logistic model (*p* < 0.001). The model obtained explains about 17% of the variance (R^2^_Naguelkerke_ = 0.169) and presents an accuracy of 63.2% which is compared with 50% obtained in the null model. Table 2 shows the regression coefficients obtained with the corresponding adjusted odds ratios (OR) and their confidence intervals (CI).

Regarding parental related factors, it was found that (1) for each maternal year increase, the probability of having a cleft child decreases 0.9 (OR = 0.903) and (2) for each BMI unit (kg/m^2^) increase, the probability of having a cleft child increases 1.14 (OR = 1.14). The child-birth data showed that for each mass unit (kg) increase at birth, the probability of having a cleft child decreases 0.4 (OR = 0.435). The area under the ROC curve for the probabilities obtained by the logistic model was 0.699 (IC95% [0.637; 0.761], *p* < 0.001).

No association between maternal age and BMI index on the risk of having a child with a cleft was found (*p* = 0.573).

The final sample of OC group consisted of 85 unilateral cleft lip and palate, 26 bilateral cleft lip and palate, 19 cleft palate, and 3 cleft lip. Table 3 presents descriptive statistics concerning phenotype of cleft. It was found that maternal BMI affects the risk of having cleft in the four clefts types (*p* = 0.023) with a statistical difference in cleft lip (Table 4).

## 4. Discussion

The purpose of this study was to evaluate the effect of parental age and mothers’ BMI on the risk of having a child with OC. Possible interaction of mother risk factors is also investigated. Moreover, birth data of cleft children were analyzed to assess the differences between patients with OC and healthy controls. Of the six study variables, three variables (maternal age, maternal body mass index, and birth weight) found statistically significant differences using the logistic model (*p* < 0.001).

The current study suggests that for each maternal year increase, the probability of having a cleft child decreases 0.9 (OR = 0.903) but no statistical differences were found regarding paternal age. The association between the risk of having cleft and parental age does not have a consensus in the literature [27,28,29]. Herkrath et al. suggested that mothers 35 years of age or older and parents 40 years of age or older had an increased risk of 20% and 58% of having a child with a cleft, respectively [27]. In contrast, Carvalho et al. did not find an association between maternal age and orofacial clefts (*p* = 0.747) in all age groups study, including mothers 35 years of age or older [28]. Nevertheless, some studies reported that the risk of having a cleft is also related to the interaction of the age of both parents [30,31]. Berg et al. showed that the risk of having cleft lip only increases when the age of both parents was high [31].

A positive association between BMI and orofacial cleft was also found: for each BMI unit (kg/m^2^) increase, the probability of having a cleft child increases 1.14 (OR = 1.14). These findings are consistent with the results of three meta-analyses that found an association between overweight women and orofacial clefts with a similar odds ratio for cleft palate and for unilateral cleft lip and palate [32,33,34]. However, the main limitation of these studies is that they did not analyze the interaction of BMI in cleft lip and bilateral cleft lip and palate. The present study proved that BMI affects the risk of having cleft in the four cleft phenotypes (*p* = 0.023) with a statistical difference in cleft lip (Table 4). A cohort study from United Kingdom, investigating the association between BMI and the majority of structural congenital anomalies, included three types of cleft (cleft lip; cleft lip and palate; and cleft palate) and found that only the cleft lip shows a statistical difference (aOR = 3.71, 95% CI: 1.05, 13.10; *p* = 0.04) [35]. The mechanism that explains how obesity acts as teratogenic factor has not been fully elucidated. Still, some authors suggested a possible explanation based on the relationship between obesity and gestational diabetes since hyperglycemia can modify the fetal expression of developmental genes, such as bone morphogenetic protein 4 [34].

Despite the average associations regarding maternal age and maternal BMI, no exploration of possible interaction in both risk factors has been done. Thus, this is the first study that intends to verify this relation, revealing that no association can be established between these two factors (*p* = 0.573). This result is unexpected because ageing is associated with an increase in abdominal white adipose tissue and fat deposition in skeletal muscle, which significantly affect insulin sensitivity [36]. Moreover, changes in gametes through life due to environmental exposures or chromosomal alterations and increased permeability to teratogenic agents in the placenta in older mothers may also increase the risk of having a child with OC [37].

Regarding child birth data (weight, length, and head circumference), all variables presented a lower mean in the cleft group compared with non-cleft individuals. However, only birth weight found statistical differences using the logistic model (*p* < 0.001), suggesting that for each increase in mass unit (kg) at birth, the probability of having a cleft decreases 0.4 (OR = 0.435). This finding is consistent with the Wyszynski et al. study, which verified that cleft palate patients with or without lip involvement had slightly lower birth weight (mean difference 159 and 197, respectively) [23]. Becker et al. also showed that patients with cleft lip with or without palate involvement were lighter and shorter than control subjects [22]. These authors attempted to hypothesize several explanations for the lower weight in cleft patients at birth: (1) incomplete development of facial tissues; (2) environment factors associated with the risk of having cleft (such as smoking and maternal dietary intake) may slow the rate of fetal development [22,23]. Despite this, children with cleft palate with or without cleft lip presented spontaneous recovery that starts at approximately 5 months of age. In addition, cleft lip children showed similar growth to healthy children [38].

The main limitation of the present study was the analysis of the different types of orofacial clefts because the division of data into subgroups results in very small numbers, especially in cleft lip where the prevalence is lower. This may explain why only BMI showed differences statically significant among the different types of cleft studied. Additionally, unmeasured confounding factors cannot be excluded even though the model obtained presents an accuracy of 63.2%. This study has several strengths compared with the published literature: (1) both groups had similar socio-economic backgrounds; (2) controls (non-cleft group) were individually matched to each case regarding age and sex; (3) a geneticist participated in the diagnosis of the cleft group, avoiding including syndromic patients; and (4) data birth were recorded by medical professionals.

This study highlights the complexity in identifying the risk factors for having orofacial cleft, especially when trying to detect differences between phenotypes. In some countries all over the world, healthcare systems cannot afford treatment for cleft lip and palate. Identification of strategies to modify risk factors for non-syndromic OC (such as being overweight) is the first step toward primary prevention [39]. As strong evidence of some of the prevention factors is still lacking, the next reasonable step for research might be the observational studies that consider the interactions of several risk factors, namely maternal family history of diabetes.

## 5. Conclusions

In this homogenous population-based study, maternal body mass index and maternal age was found to affect the risk of having a cleft child. However, the association of these two factors does not increase the risk. In the children’s initial data, the cleft group found a higher risk of having a lower birth weight but no relation was found regarding length and head circumference. This trial and further studies will allow the incorporation of the findings into preconception counseling health care programs for women of reproductive age.

## Figures and Tables

**Table 1:** Descriptive analysis by each group.

Variables	Control (133)	Orofacial Cleft (133)	*p*
Sex (M/F)	85/48 (63.9%/36.1%)	85/48 (63.9%/36.1%)	1.000 ^#^
Maternal age (years) ^a^	31 (4.2) 28.0/34.0	28 (5.6) 25.0/32.0	<0.001 ^§^
Paternal age (years) ^a^	33 (4.9) 29.0/36.0	31 (6.3) 27.0/35.0	0.016 ^§^
Maternal BMI (kg/m^2^) ^a^	23 (3.9) 20.8/25.1	25 (4.3) 21.9/28.3	<0.001 ^§^
Birth Length (cm) ^a^	49 (2.1) 48.0/50.0	48 (2.9) 47.0/50.0	0.519 ^§^
Birth Weight (kg) ^a^	3 (0.5) 3.0/3.6	3 (0.5) 2.8/3.4	0.033 ^§^
Birth Head circumference (cm) ^a^	35 (2.2) 33.5/35.0	34 (2.3) 33.5/35.0	0.457 ^§^

^a^ mean (standard deviation) 25–75 percentiles; ^#^ Fisher’s exact test; ^§^ Mann-Whitney, M-male, F-female, BMI- body mass index.

**Table 2:** The regression coefficients for maternal age, maternal BMI, and weight at birth.

Variables	B	*p*	OR	CI95%
Maternal age (years)	−0.102	<0.001	0.903	[0.856; 0.953]
Maternal BMI (kg/m^2^)	0.131	<0.001	1.140	[1.068; 1.216]
Birth Weight (kg)	−0.833	0.003	0.435	[0.253; 0.746]
	2.477	0.062	11.904	

B—regression coefficient; OR—odds ratio; CI—confidence interval.

**Table 3:** Descriptive statistics concerning phenotype of cleft.

Variables	Unilateral Cleft Lip and Palate (*n* = 85)	Bilateral Cleft Lip and Palate (*n* = 26)	Cleft Palate (*n* = 19)	Cleft Lip (*n* = 3)	*p*
Maternal age (years) ^a^	28 (5.4) 25.0/31.0	28 (5.8) 24.0/31.0	30 (5.8) 26.0/37.0	30 (9.1) 23.0/40.0	0.4271 ^§^
Paternal age (years) ^a^	31 (6.2) 27.0/34.0	32 (6.9) 28.0/34.0	34 (5.4) 29.0/38.0	27 (3.2) 25.0/31.0	0.121 ^£^
Maternal BMI (kg/m^2^) ^a^	25 (4.2) 22.3/27.6	25 (5.0) 20.3/29.1	25 (3.6) 21.8/26.4	33 (1.7) 30.8/34.2	0.023 ^£^
Birth Length (cm) ^a^	48 (2.7) 47.0/50.0	48 (2.6) 47.0/50.0	49 (4.3) 48.0/51.0	48 (1.2) 47.0/49.0	0.452 ^§^
Birth Weight (kg) ^a^	3 (0.5) 2.8/3.4	3 (0.5) 2.9/3.5	3 (0.4) 2.9/3.4	3 (0.7) 2.2/3.6	0.505 ^£^
Birth Head circumference (cm) ^a^	34 (1.7) 33.0/35.0	34 (1.2) 33.5/35.0	36 (4.3) 33.5/36.0	33 (2.6) 30.0/35.0	0.702 ^§^

^a^ mean (standard deviation) 25–75 percentiles; ^§^ Kruskal-Wallis; ^£^ ANOVA.

**Table 4:** Correlation between maternal BMI and cleft phenotype.

Phenotype of Cleft	*p*
Unilateral cleft lip and palate vs. bilateral cleft lip and palate	*p* = 0.990
Unilateral cleft lip and palate vs. cleft palate	*p* = 0.947
Unilateral cleft lip and palate vs. cleft lip	*p* = 0.016
Bilateral cleft lip and palate vs. cleft palate	*p* = 0.995
Bilateral cleft lip and palate vs. cleft lip	*p* = 0.016
Cleft palate vs. cleft lip	*p* = 0.014

## Data Availability

The data presented in this study are available on request from the corresponding author.
